# Genes for cooperation are not more likely to be carried by plasmids

**DOI:** 10.1098/rspb.2023.2549

**Published:** 2024-02-28

**Authors:** Anna E. Dewar, Laurence J. Belcher, Thomas W. Scott, Stuart A. West

**Affiliations:** Department of Biology, University of Oxford, Oxford OX1 3SZ, UK

**Keywords:** horizontal gene transfer, social evolution, comparative genomics, phylogenetic comparative methods

## Abstract

Cooperation is prevalent across bacteria, but risks being exploited by non-cooperative cheats. Horizontal gene transfer, particularly via plasmids, has been suggested as a mechanism to stabilize cooperation. A key prediction of this hypothesis is that genes which are more likely to be transferred, such as those on plasmids, should be more likely to code for cooperative traits. Testing this prediction requires identifying all genes for cooperation in bacterial genomes. However, previous studies used a method which likely misses some of these genes for cooperation. To solve this, we used a new genomics tool, SOCfinder, which uses three distinct modules to identify all kinds of genes for cooperation. We compared where these genes were located across 4648 genomes from 146 bacterial species. In contrast to the prediction of the hypothesis, we found no evidence that plasmid genes are more likely to code for cooperative traits. Instead, we found the opposite—that genes for cooperation were more likely to be carried on chromosomes. Overall, the vast majority of genes for cooperation are not located on plasmids, suggesting that the more general mechanism of kin selection is sufficient to explain the prevalence of cooperation across bacteria.

## 1. Introduction

Cooperation appears to play a key role in the growth and success of many bacteria [1–3]. Bacteria produce and secrete a range of molecules that provide benefits to the local population of cells, and therefore act as cooperative ‘public goods’. Examples include iron-scavenging siderophores and enzymes that can break down host defences [4–7]. The problem with such cooperation is that the benefit of producing public goods is potentially shared with ‘cheat’ cells that do not produce the public good, which could lead to cooperation being unstable [8]. A likely solution is that the clonal growth of bacteria means that public goods are shared mainly with relatives (clonemates) that also carry the gene for cooperation, an example of kin selection [9].

Horizontal gene transfer has been suggested as another mechanism to stabilize cooperation in bacteria [10–24]. Horizontal transfer of genes for cooperation could increase relatedness at those loci and prevent invasion by non-cooperative cheats. Horizontal gene transfer could even lead to high relatedness at the loci for cooperation between cells that are not genetically related across the rest of the genome. This possibility has been explored theoretically in particular for plasmids, which are extra-chromosomal sequences found across bacteria, and which are often capable of transferring, along with all the genes they carry, to other cells [10–14].

A key prediction of this hypothesis is that genes for cooperation should be overrepresented on more mobile parts of the genome, such as plasmids compared with chromosomes [19,25]. If cooperation is favoured by horizontal gene transfer, then genes for cooperative traits are more likely to be maintained if they are on plasmids, or can be preferentially moved onto plasmids. Plasmids usually carry far fewer genes than chromosomes, suggesting there is likely to be some constraints associated with moving extra genes onto a genome's plasmids. Therefore, to control for the difference in chromosome and plasmid size, the prediction of the hypothesis is expressed as a relative proportion of genes: all else being equal, if cooperation benefits from horizontal gene transfer such as via plasmids, a higher proportion of plasmid genes should code for cooperative traits compared with the chromosome (i.e. overrepresented on plasmids).

Our recent comparative genomics analysis of 51 species of bacteria did not find support for the horizontal gene transfer hypothesis [26]. We tested the hypothesis by examining the genomic location of genes which coded for proteins that are secreted by bacteria into the extracellular space (genes coding for extracellular proteins). These proteins are likely to act as public goods because they will often diffuse away from the producing cell, and so any benefit of their function is shared by neighbouring cells. In contrast to the predictions of the horizontal gene transfer hypothesis, we found that: (i) genes coding for extracellular proteins were not overrepresented on plasmids compared with chromosomes, (ii) plasmids with a higher mobility did not carry more genes coding for extracellular proteins [26].

However, there are potential problems with using genes coding for extracellular proteins as a method for identifying genes for cooperation. Extracellular proteins that act as public goods are among the simplest kind of cooperative behaviour in bacteria, because one gene codes for one protein which is secreted out of the cell to act as a cooperative public good. However, other genes code for cooperative traits in more complex ways, such as by coding for a protein which combines with other proteins and molecules inside the cell before being secreted, or by catalysing a reaction that helps make the cooperative molecule. For example, iron-scavenging siderophores are secondary metabolites of a large gene cassette, with each gene coding for intracellular proteins which work together to produce the secreted siderophore molecules [27]. Genes coding for siderophores would therefore not be counted as cooperative, despite siderophores being one of the most studied cooperative traits in bacteria [28]. Consequently, analyses considering only genes for extracellular proteins are likely to miss a number of genes involved in cooperation.

Additionally, our previous analysis was based on the genomic data available in 2019, which was only 51 species, and biased towards human pathogens [26]. Since then, the number of complete prokaryotic genomes in the RefSeq database has more than doubled, meaning there is now potential to examine a much wider and more representative range of bacterial species [29].

We addressed these problems by conducting a comparative genomics analysis using a new tool for identifying a broad range of genes for cooperation (SOCfinder), not just those coding for extracellular proteins [30]. SOCfinder comprises three modules which identify genes for extracellular proteins, genes with a cooperative functional annotation and genes which are part of a cooperative secondary metabolite cluster. In addition, we were able to expand the dataset to 146 species, almost three times as many species as in the previous study [26]. We used phylogeny-based statistical methods to control for non-independence of species, and also for any unevenness in the taxonomic distribution of studied species. We tested whether genes for cooperation were more likely to be carried on plasmids compared with chromosomes, and also examined whether this differed for each of three broad kinds of genes for cooperative traits.

## 2. Methods

### (a) Selecting species and downloading genomes

We included species in our analysis if they had at least 10 complete genomes in the RefSeq database, and that at least 10 of those genomes had at least one plasmid sequence in their assembly. For all species meeting the criteria, we then downloaded all genomes available in RefSeq up to a maximum of 100. For the species which had more than 100 genomes available, we randomly selected 100 genomes for further analysis. We then downloaded the RefSeq genomes using the National Center for Biotechnology Information (NCBI) Datasets conda package (version 15.5.0) (https://www.ncbi.nlm.nih.gov/datasets). Overall, our dataset included a total of 4648 genomes across 146 bacterial species.

### (b) Using SOCfinder to identify genes for cooperative traits

SOCfinder finds genes for cooperative traits using three modules which each search for different kinds of genes: (i) genes for extracellular proteins, (ii) genes with a ‘cooperative’ functional annotation, (iii) genes which are part of a ‘cooperative’ secondary metabolite gene cluster [30]. The results of these three modules are then combined, avoiding any double counting if the gene is flagged by more than one module, and a list of all genes coding for cooperative traits found in the genome is provided.

We ran SOCfinder (version 1.0.1) (https://github.com/lauriebelch/SOCfinder) with default parameters on all our genomes [30]. We then matched the list of genes for cooperation found by SOCfinder to each genome's chromosome(s) or plasmid(s). We did this for the consensus list which combines results of all three modules, and also for each of the modules separately. This was so we could compare whether considering different kinds of genes for cooperation influenced our results. We used a number of python, bash and R scripts during our selection and downloading of genomes, and running SOCfinder, all of which are available at: https://github.com/AnnaEDewar/Plasmid_SOCfinder.

### (c) Phylogeny

We used two phylogenies to control for any phylogenetic non-independence between species. First, we generated a supertree phylogeny of the 146 species in our dataset, using methods as described in Dewar *et al.* [26]. We used a recently published maximum-likelihood tree generated with ribosomal protein data as the basis for our phylogeny [31]. We used the R package ‘ape’ to identify all branches that matched either a species or a genus in our dataset [32]. In cases where we had multiple species within a single genus, we used the R package ‘phytools’ to add these species as additional branches in the tree [33]. We used published phylogenies from the literature to add any within-genus clustering of species' branches (details in electronic supplementary material S2). We used this phylogeny for our main statistical analyses (electronic supplementary material, figure S4).

Second, to check the robustness of our results, we also used the Genome Taxonomy Database (GTDB) bacterial reference tree as an alternative phylogeny for the genomes in our dataset [34]. The structure of the tree is inferred using alignments of 120 conserved bacterial concatenated proteins, which is then used to infer species clusters [35]. We downloaded the GTDB tree and associated metadata for all genomes used to make the tree, and used these to identify whether each of our genomes had a representative in the tree, and if so, identified each genomes’ species cluster and associated representative genome (v. 214.1). We were able to do this for 4364 of our genomes; we could not locate 284 genomes within the GTDB metadata table, and so these were removed before further analysis using the GTDB tree. We then produced a subset of the GTDB tree with only tips that represented one or more of our genomes, producing a tree with 284 GTDB species clusters, ranging from 1 genome to 220 genomes corresponding to each cluster (electronic supplementary material, figure S5). We analysed trends across all genomes in all species clusters, and also analysed a subset of species clusters which had at least 10 genomes in our dataset.

### (d) Data and statistical analyses

When comparing plasmids and chromosomes, it is important to do this at the level of individual genomes, which is where selection will be acting, rather than grouping all genes together from multiple genomes and/or species. This genome-level approach also allowed us to control for potential confounding factors, such as different numbers of genes across species and non-independence of species due to common ancestry.

Therefore, for each genome we calculated the proportion of genes coding for cooperative traits on both their chromosome(s) and their plasmid(s). We then analysed whether these proportions were significantly different. We used two methods to control for the fact that proportion data is not normally distributed: arcsine square root transformation and logit transformation.

For both, we calculated the difference in plasmid and chromosome proportions for each genome. We did this by subtracting the chromosome proportion from the plasmid proportion (P–C). Therefore, if the difference in proportion is greater than 0, genes for cooperative traits are overrepresented on plasmids, while if the difference in proportion is less than 0, genes for cooperative traits are overrepresented on chromosomes. We used a Bayesian mixed-effects model, with phylogenetic similarity as a random effect, to test whether the difference in proportion was significantly different from zero. We did this both using the mean difference for each species, and also considering each genome as a data point (where we also included species as a random effect).

As a third way to control for the non-normality of proportion data, we also conducted a binomial analysis, where we examined how the number of cooperative genes compared with non-cooperative genes differed between plasmids and chromosomes (electronic supplementary material, S1; §1.1.4).

For all Bayesian mixed-effects models we used the R package MCMCglmm with phylogeny as a random effect [36]. A MCMCglmm (Markov Chain Monte Carlo generalized linear mixed models) is a type of Bayesian statistical model often used to control for phylogenetic regressions. The evolutionary history of bacterial species could mean that closely related species have more similar genomes, regardless of other factors [37]. Consequently, we controlled for the phylogenetic relationships between species by setting phylogeny as a random effect in all our models. For our results we have reported the pMCMC value, which for simplicity can be interpreted as one interprets a ‘*p*-value’. The full results for all models can be found in electronic supplementary material S1, and code for all models is available at https://github.com/AnnaEDewar/Plasmid_SOCfinder.

## 3. Results

### (a) Genes for cooperation are not more likely to be carried on plasmids

**Figure 1.**
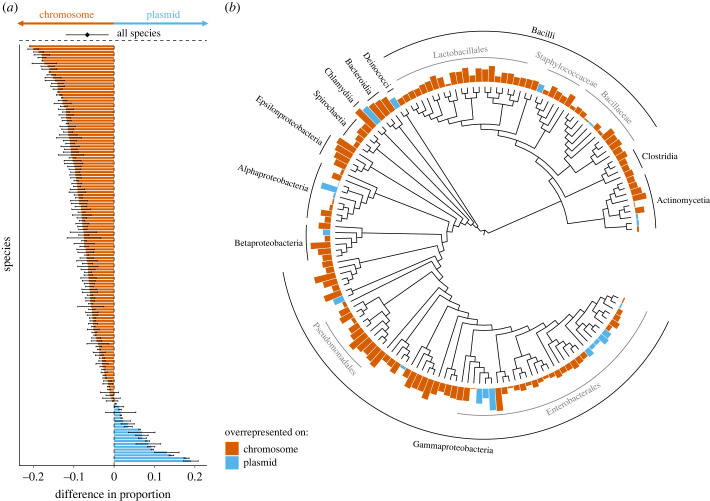
Plasmid genes are not more likely to code for cooperative traits. (*a*) For each species we calculated the mean difference between plasmid(s) and chromosome(s) in the proportion of genes coding for cooperative traits, which is displayed as a bar for each species. Species with a blue bar have a higher proportion of genes coding for cooperative traits on their plasmid(s), while species with a red bar have a higher proportion of genes coding for cooperative traits on their chromosome(s). For each species, the error bars indicate the standard error of the mean. Electronic supplementary material, figure S1, provides an alternate version which includes species names on the *y*-axis. The black dot and error bars indicate the mean difference and the 95% Credible Interval given by a MCMCglmm analysis across all 146 species, controlling for phylogenetic similarity and sample size. We arcsine square root transformed proportion data before calculating the difference; electronic supplementary material, figure S2, is an alternate version of the same plot but with the difference calculated from logit transformed proportions. (*b*) The bars in panel (*a*) are plotted onto our supertree phylogeny of all 146 species, with higher bars corresponding to species with a greater difference between plasmids and chromosomes, and colour indicating whether plasmids (blue) or chromosomes (red) have a greater proportion. Bacterial taxonomic classes annotated in black and additional taxonomic groups in grey. Overall, plasmid genes were significantly less likely to code for cooperative traits compared with chromosome genes.

Contrary to the prediction of the horizontal gene transfer hypothesis, we found that plasmid genes were not more likely to code for cooperative traits. In fact, we found the opposite: plasmid genes were *less* likely to code for cooperative traits than chromosome genes (figure 1). Specifically, we found the difference in the proportion of genes coding for cooperative traits between plasmids and chromosomes was significantly less than zero, indicating that the plasmid proportion was lower than the chromosome proportion across species (MCMCglm; arcsine: posterior mean = −0.066, 95% CI = −0.12 to −0.013, pMCMC = 0.017*, figure 1, electronic supplementary material, table S1; logit: posterior mean = −0.300, 95% CI = −0.556 to −0.032, pMCMC = 0.028*, electronic supplementary material, figure S2, table S2).

We tested the robustness of this result by using an alternative phylogeny, from the GTDB, which included some different species definitions across our genomes [34]. We found the same result: plasmid genes were less likely to code for cooperative traits compared with chromosome genes (MCMCglmm; arcsine: posterior mean = −0.061, 95% CI = −0.115 to −0.008, pMCMC = 0.0283*, electronic supplementary material, table S3; logit: posterior mean = −0.286, 95% CI = −0.536 to −0.031, pMCMC = 0.034*, electronic supplementary material, table S4).

We also found that plasmids were less likely to carry genes for cooperative traits when we analysed the data with a binomial model, which compared the number of cooperative and non-cooperative genes on plasmids and chromosomes across, but not within, genomes (Binomial MCMCglmm; posterior mean = −0.105, 95% CI = −0.119 to −0.091, pMCMC < 0.001***, electronic supplementary material, table S7).

### (b) Chromosomes carry the vast majority of genes for cooperation

We followed previous studies by focusing on comparing proportions of plasmid and chromosome genes that are cooperative, rather than the absolute number [19,25,26]. However, we can also examine the data in a number of different ways.

In our dataset, an average bacterial genome contained 2835 (SE = ±106) chromosome genes and 208 (SE = ±30) plasmid genes (MCMCglmm; electronic supplementary material S1). Overall, the average proportion of genes in a genome which were carried on plasmid(s) was 4.2% (95% CI = 1.3–9.1%), when we first calculated the proportion for each genome before controlling for phylogeny and sample size (electronic supplementary material, table S12).

**Figure 2.**
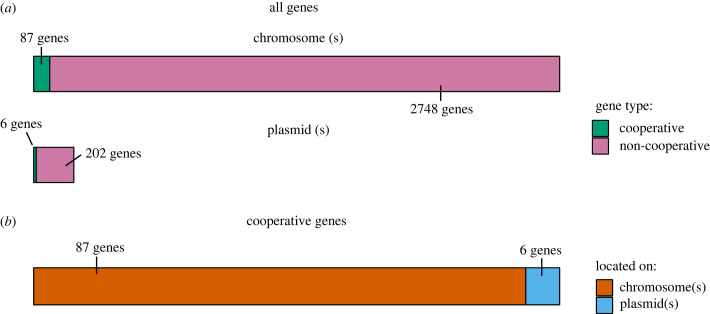
Visualization of where genes for cooperative traits are located within an average bacterial genome. (*a*) The bars indicate the average number of genes carried on a genome's chromosome(s) (upper panel), and plasmid(s) (lower panel). Each bar is split by whether the genes code for cooperative (green) or non-cooperative traits (pink). In this panel, the size of the bar is directly proportional to the number of genes. The number of genes (to the nearest gene) is annotated next to the corresponding segment. (*b*) The bar indicates the number of genes for cooperative traits carried on an average genome's plasmid(s) (blue segment) and chromosome(s) (red segment). The number of genes (to the nearest gene) is annotated next to the corresponding bar. Overall, the vast majority of genes for cooperative traits are carried by the chromosome(s).

Considering only genes for cooperative traits, there was an average of 6 (SE = ±1) genes for cooperation on plasmids and 87 (SE = ±6) genes for cooperation on chromosome(s) (MCMCglmm; electronic supplementary material S1, tables S8 and S10) (figure 2). After controlling for phylogeny and sample size, an average genome's proportion of genes that coded for cooperative traits was 2.8% (95% CI = 0.1–5.8%; electronic supplementary material, table S13). Of those genes for cooperative traits, an average of 2.1% were on plasmids (95% CI = 0.01–5.6%; electronic supplementary material, table S14).

Overall, these analyses show that bacteria carry very few of their genes for cooperative traits on plasmid(s) (average = 6 genes), despite plasmids generally carrying a total number of genes that is much greater than the total genes for cooperation in the genome (average of 208 plasmid genes per genome versus an average of 93 genes for cooperative traits per genome; figure 2). Electronic supplementary material, table S21, lists the mean number of genes for cooperative and non-cooperative traits on plasmids and chromosomes, and the mean proportion of genes which are carried on plasmids.

### (c) Influence of including other kinds of genes for cooperative traits

**Figure 3.**
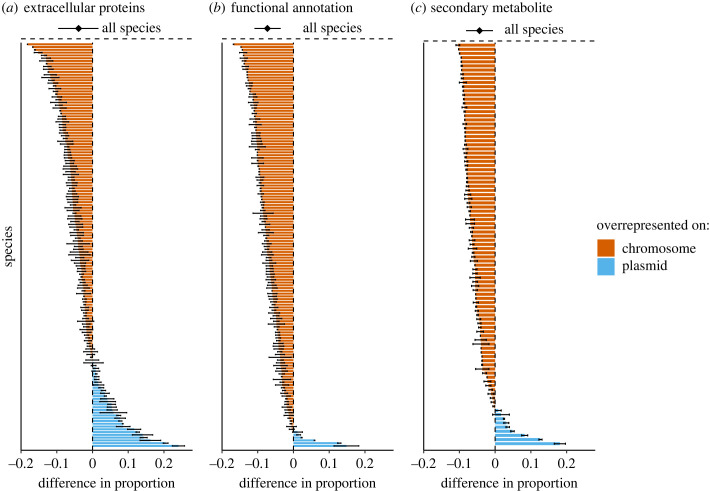
Comparison of three broad types of genes for cooperation identified by SOCfinder. As in figure 1, for each species we calculated the mean difference between plasmid(s) and chromosome(s) in the proportion of genes coding for cooperative traits, which is displayed as a bar for each species. We did this separately for the three broad categories of genes for cooperative traits identified by SOCfinder: (*a*) genes for extracellular proteins, (*b*) genes with a cooperative functional annotation, (*c*) genes which are part of a cooperative secondary metabolite cluster. In each panel, species with a blue bar have a higher proportion of genes coding for that type of cooperative trait on their plasmid(s), while species with a red bar have a higher proportion of that type of gene coding for a cooperative trait on their chromosome(s). For each species, the error bars indicate the standard error of the mean. The black dot and error bars indicate the mean difference and the 95% Credible Interval given by a MCMCglmm analysis across species, controlling for phylogenetic similarity and sample size; N = 146 species for panels (*a*) and (*b*), and N = 97 species for panel (*c*) (49 species had genomes which did not have any genes found by this SOCfinder module, so we have removed them from this plot and analysis, since there is no mathematical definition for a proportion with 0 as both the numerator and denominator). We arcsine square root transformed proportion data before calculating the difference; electronic supplementary material, figure S3, is an alternate version of the same plot but with the difference calculated from logit transformed proportions. Overall, genes for extracellular proteins were equally likely to be found on plasmid(s) and chromosome(s), while genes with a cooperative functional annotation and genes which were part of a cooperative secondary metabolite cluster were more likely to be carried on chromosome(s).

SOCfinder, the genomic tool we used to identify genes for cooperative traits, includes three modules which each use a different method to find genes for cooperative traits [30]. These three different types of genes for cooperative traits are: (i) genes coding for extracellular proteins, (ii) genes with a cooperative functional annotation, (iii) genes which are part of a cooperative secondary metabolite cluster. We compared the plasmid and chromosome proportions of genes found by each of these three modules, to examine whether our results were consistent when we considered each separately (figure 3).

We found that none of the three subsets of genes for cooperative traits identified by SOCfinder showed support for the horizontal gene transfer hypothesis (figure 3). Genes coding for extracellular proteins were equally likely to be found on plasmid(s) and chromosome(s) (MCMCglmm; arcsine: posterior mean = −0.039, 95% CI = −0.098 to 0.017, pMCMC = 0.174, non-significant (NS); logit: posterior mean = −0.113, 95% CI = −0.381 to 0.156, pMCMC = 0.372, NS; electronic supplementary material, tables S15 and S16). Genes which had a cooperative functional annotation and genes which were part of a cooperative secondary metabolite cluster were both significantly more likely to be found on chromosome(s) (MCMCglmm; Cooperative function annotation, arcsine: posterior mean = −0.073, 95% CI = −0.11 to −0.039, pMCMC < 0.001***; logit: posterior mean = −0.253, 95% CI = −429 to −0.077, pMCMC = 0.008**; electronic supplementary material, tables S17 and S18; Cooperative secondary metabolite cluster, arcsine: posterior mean = −0.044, 95% CI = −0.08 to −0.007, pMCMC = 0.0291*; logit: posterior mean = −0.11, 95% CI = −0.206 to −0.005, pMCMC = 0.0486*, electronic supplementary material, tables S19 and S20).

## 4. Discussion

We have shown, across 146 bacterial species, that genes for cooperation are not more likely to be on plasmids. Instead, we found evidence that plasmid genes are actually less likely to code for cooperative traits compared with chromosome genes (figure 1). An average bacterial genome carries only 2% of its genes for cooperation on its plasmid(s), with the remaining 98% of its genes for cooperation on the chromosome(s) (figure 2).

### (a) Where are genes for cooperation located within bacterial genomes?

Contrary to the key prediction of the horizontal gene transfer hypothesis, we found the opposite – plasmids had a significantly lower proportion of genes for cooperation than chromosomes. This result was driven by genes found by the two additional modules of SOCfinder: genes with a cooperative functional annotation and genes which were part of a cooperative secondary metabolite cluster were both more likely to be found on chromosomes (figure 3). The genes found by these two additional modules generally code for more complex cooperative traits compared with genes for extracellular proteins. We recently found that plasmid genes have a consistently lower complexity compared with chromosomal genes, where complexity was measured by the number of connections each gene had within the genome's protein–protein interaction network [38]. This lower complexity on plasmids could explain our result that chromosomes carry proportionally more of these more complex cooperative traits. Carriage on chromosomes could be more likely to be favoured for relatively more complex traits, because carriage on a plasmid could risk the breakup of the gene cassette or be non-functional and/or metabolically disruptive if the plasmid was transferred into a new recipient or over-expressed due to high copy number [39,40].

The carriage of certain genes on plasmids could be favoured for reasons other than horizontal gene transfer [26,41]. One potential benefit is that plasmids usually exist as multiple copies in the cell, meaning plasmid genes will, on average, have a higher expression than genes on the single copy chromosome(s). This could be important for traits like secreted virulence factors and antibiotic resistance mechanisms, where the strength of the phenotype will be directly related to the quantity of the effector molecule produced. For example, bacteria carrying a gene coding for a secreted beta-lactamase on a multi-copy plasmid had a higher level of resistance to beta-lactam antibiotics compared with those carrying the same gene on a chromosome [22]. These benefits could explain why Dewar *et al.* found that species which were broad host-range pathogens were most likely to have genes for extracellular proteins overrepresented on their plasmids [26]. Taken together, even when genes for cooperative traits are carried on plasmids, it could be for a reason other than the plasmid's ability to transfer.

Methods to find genes for cooperative traits could disproportionately miss those carried on plasmids, because plasmid genes tend to be less well annotated. However, we have several reasons why we think this is unlikely to be driving our results. First, when we looked at only genes for extracellular proteins, which are identified by the presence of a highly conserved signal peptide sequence and so should be unaffected by gene annotation, we found no difference in plasmid and chromosome proportion of genes for cooperation (figure 3*a*).

Second, most of the focus in the literature has been on the proportion of genes for cooperation on plasmid(s) and chromosome(s), because plasmids usually carry far fewer genes than the chromosome(s) [19,25,26]. In our dataset, the average bacterial genome carries only 4.6% of its genes on plasmid(s). However, comparing the proportion to control for this imbalance in number of genes ignores something often overlooked: the imbalance is itself evidence against a major role of horizontal gene transfer via plasmids in the maintenance of cooperation.

Third, the previous comparative genomics study across 51 species found that plasmid mobility, defined as the extent to which a plasmid was able to mobilize via conjugation, did not correlate overall with the proportion of a plasmid's genes which coded for extracellular proteins [26]. We have not considered plasmid mobility in this study, and it offers an alternate method for examining why genes are carried on plasmids.

In terms of absolute number, instead of proportion, we found that the vast majority (98%) of genes for cooperative traits are carried on the chromosome(s) (figure 3). If we were missing a few plasmid genes for cooperation due to a lower annotation quality, we would have needed to miss an average of 81 genes for cooperation on plasmids for there to be a higher absolute number of genes for cooperation on plasmids compared with chromosomes. However, for this to be the case, more than 41% (87/208) of all plasmid genes across bacterial genomes would have to code for a cooperative trait, compared with only 2.8% of the genome as a whole. We think this is very unlikely. Instead, what this imbalance in number of genes suggests is that very few genes for cooperation in bacteria ever benefit from plasmid transfer, and yet cooperation is highly prevalent and stable across bacteria. Something else other than horizontal gene transfer must maintain cooperation for the approximately 98% of genes for cooperative traits carried on chromosomes.

### (b) Why is cooperation not favoured by horizontal gene transfer?

Our results support recent theory which suggested that horizontal gene transfer does not appreciably favour or stabilize cooperation [26,42]. Older theory had focused on the invasion of cooperation, and found that this could be facilitated by plasmids [10,12,13,19–21]. However, plasmids can facilitate the invasion of any gene, and not just cooperation. In addition, when the potential for cheating as well as cooperative plasmids was allowed for, it was found that horizontal gene transfer did not appreciably help maintain cooperation [26,42]. Cooperation tends to only be favoured on plasmids in the same conditions where it is favoured on chromosomes.

An exception, where cooperation can be preferentially favoured on plasmids, is if the rate of plasmid transfer is high and the rate of plasmid loss is intermediate [26,42]. However, these conditions also lead to a low rate of plasmid carriage, and so plasmids can only have a small effect on the level of cooperation. Put simply, plasmids either evolve to be rare and more cooperative, or common and not more cooperative. Consequently, theory predicts that the overall influence of plasmid transfer on the level of cooperation in bacterial populations will be low or negligible [42].

More generally, theory and empirical work examining this hypothesis has tended to assume that there are no fundamental differences between plasmids and chromosomes, other than their ability to transfer. However, it is now clear that there are many features of plasmids which could reduce the suitability of directly comparing their gene content to bacterial chromosomes [41]. First, plasmids can exist in many copies per cell, which could lead to genetic dominance effects and potentially impact dynamics of plasmid-carried cooperative loci within cells [43]. Second, plasmids carry ‘backbone’ genes which allow them to replicate and in the case of mobilizable and conjugative plasmids, transfer via bacterial conjugation [15]. The presence of these genes will constrain the maximum proportion of plasmid genes that can code for traits such as cooperation. While the hundreds of essential genes that bacterial chromosomes must carry will also impose a similar constraint on the maximum proportion of chromosomal genes coding for cooperative traits, it is unclear how similar the proportion of these core plasmid and chromosome genes are. Third, plasmids will be under selection to increase their own transmission, which could include reducing costs to their hosts. Consequently, plasmids could be constrained by the number of genes they carry and the metabolic cost associated with those genes. This could lead to conflict between selection for plasmid carriage of a gene for cooperation compared with selection at the plasmid level to reduce gene number [39,44].

### (c) What favours cooperation in bacteria?

Kin selection provides a simple and widely applicable explanation for cooperation in bacteria, without needing to invoke a special role of horizontal gene transfer [9]. The clonal growth of bacteria means that individuals are more likely to be near relatives (kin), who would also carry the genes for cooperative traits. Consequently, any benefits of a secreted public good molecule would be shared with relatives who are also producing that molecule [45]. By contrast, non-producers will be growing with other non-producers, and so will be less able to benefit from (cheat) the public goods produced by other cells.

The kin selection hypothesis has been supported by experimental evolution, population genetics and across-species comparative studies. Experiments on a number of bacterial species have shown that the production of public goods is maintained when species are cultured at a high relatedness, but lost when cultured at a low relatedness [4,28,46–48]. Population genetic studies on *Pseudomonas aeruginosa* and *Bacillus subtilis* have shown signatures (footprints) of selection at the genomic level that are expected from kin selection for cooperation [49,50]. Specifically, genes controlling cooperative traits showed higher polymorphism, greater divergence and were more likely to harbour deleterious mutations, relative to genes for non-cooperative private traits. Comparative studies have found that species which form groups where relatedness is likely to be higher show higher levels of cooperation, as measured by the occurrence of altruistic helping cells, frequency of genes for cooperative traits or aid provided to insect hosts [51–53].

## 5. Conclusion

Combining our findings with both previous analyses and recent theoretical modelling, there is now convincing evidence that plasmid transfer does not specifically favour cooperation in bacteria [26,42]. We are not saying that horizontal gene transfer has no influence on the evolution of genes for cooperative traits in bacteria. Horizontal gene transfer is highly prevalent in bacteria, and therefore will likely influence many aspects of how genes for cooperative traits are maintained in and spread though populations. However, it will also influence the spread of many genes for non-cooperative traits in the same or analogous ways. Many kinds of genes in bacteria are transferred via horizontal gene transfer, allowing for the rapid spread of traits such as antibiotic resistance and virulence factors, irrespective of whether they are cooperative. Similarly, there are many other factors, such as gene complexity, that can influence where genes are more likely to be carried, irrespective of whether they are for cooperative traits [38].

## Data Availability

Data is available to download from GitHub at: https://github.com/AnnaEDewar/Plasmid_SOCfinder and from Dryad Digital Repository: https://doi.org/10.5061/dryad.c866t1gd8 [54]. Code availability: code for all analyses is available at: https://github.com/AnnaEDewar/Plasmid_SOCfinder. Full methods and results are provided in electronic supplementary material [55].
